# Synthesis and Characterization of pH-Sensitive Inulin Conjugate of Isoniazid for Monocyte-Targeted Delivery

**DOI:** 10.3390/pharmaceutics11110555

**Published:** 2019-10-28

**Authors:** Franklin Afinjuomo, Thomas G. Barclay, Ankit Parikh, Rosa Chung, Yunmei Song, Gayathri Nagalingam, Jamie Triccas, Lixin Wang, Liang Liu, John D. Hayball, Nikolai Petrovsky, Sanjay Garg

**Affiliations:** 1 School of Pharmacy and Medical Sciences, University of South Australia, Adelaide, South Australia 5001, Australia; olumide.afinjuomo@mymail.unisa.edu.au (F.A.); tom.barclay@unisa.edu.au (T.G.B.); ankit.parikh@unisa.edu.au (A.P.); rosa.chung@unisa.edu.au (R.C.); may.song@unisa.edu.au (Y.S.); lixin.wang@mymail.unisa.edu.au (L.W.); liang.liu@unisa.edu.au (L.L.); john.hayball@unisa.edu.au (J.D.H.); 2 Tuberculosis Research Program, Centenary Institute, University of Sydney, Sydney, NSW 2006, Australia; gnagalingam463@gmail.com (G.N.); jamie.triccas@sydney.edu.au (J.T.); 3 Vaxine Pty. Ltd., Adelaide, SA 5042, Australia; nikolai.petrovsky@flinders.edu.au; 4 College of Medicine and Public Health, Flinders University, Adelaide, SA 5042, Australia

**Keywords:** inulin microparticles, isoniazid, tuberculosis, intracellular delivery

## Abstract

The use of particles for monocyte-mediated delivery could be a more efficient strategy and approach to achieve intracellular targeting and delivery of antitubercular drugs to host macrophages. In this study, the potential of inulin microparticles to serve as a drug vehicle in the treatment of chronic tuberculosis using a monocytes-mediated drug targeting approach was evaluated. Isoniazid (INH) was conjugated to inulin via hydrazone linkage in order to obtain a pH-sensitive inulin-INH conjugate. The conjugate was then characterized using proton nuclear magnetic resonance (^1^HNMR), Fourier transform infrared spectroscopy (FTIR) as well as in vitro, cellular uptake and intracellular *Mycobacterium tuberculosis* (Mtb) antibacterial efficacy. The acid-labile hydrazone linkage conferred pH sensitivity to the inulin-INH conjugate with ~95, 77 and 65% of the drug released after 5 h at pH 4.5, 5.2, and 6.0 respectively. Cellular uptake studies confirm that RAW 264.7 monocytic cells efficiently internalized the inulin conjugates into endocytic compartments through endocytosis. The intracellular efficacy studies demonstrate that the inulin conjugates possess a dose-dependent targeting effect against Mtb-infected monocytes. This was through efficient internalization and cleavage of the hydrazone bond by the acidic environment of the lysosome, which subsequently released the isoniazid intracellularly to the Mtb reservoir. These results clearly suggest that inulin conjugates can serve as a pH-sensitive intracellular drug delivery system for TB treatment.

## 1. Introduction

Tuberculosis (TB) is a chronic bacterial infection caused by the intracellular pathogen *Mycobacterium tuberculosis* (Mtb). TB is a major threat to public health and currently affects about one-third of the global population [1]. The latest World Health Organisation report 2018 on TB gives insight into the mortality and morbidity with 1.3 million estimated deaths and another 10.0 million estimated new cases of TB [2]. From the latest data, undeniably TB is now the number one cause of death exceeding even human immunodeficiency virus infection (HIV) and acquired immune deficiency syndrome (AIDS) [2]. The chemotherapy and management of TB are complicated by many factors such as multidrug resistance, multiple regimens, long treatment duration and the deadly synergy between HIV and TB [3,4,5,6,7,8].

Mtb uses the alveolar macrophages as an exclusive dwelling place [9]. Apart from residing in the macrophages, Mtb further uses infected monocytes as cellular tank/storage and vehicles (Trojan horse) to spread this infection to other organs as well as other parts of the body [10,11]. In addition to this, the Mtb has adapted several evading mechanisms coupled with other strategies to ensure intracellular survival by controlling the phagosomes maturation [12,13], blockage of phagocytosed materials to the lysosome and evasion of elimination by the host monocytic system [14]. To further complicate the issue, Mtb infection leads to the formation of granulomas, which ultimately become sites for replication. Furthermore, the unique but complex mycobacterial cell-wall scaffold creates a difficult barrier for the delivery of conventional TB drugs to the pathogen. Consequently, the Mtb pathogen manipulates the alveolar macrophages of the host, resulting in chronic, long-term infections [15]. This survival mechanism necessitates the extended use of TB drugs (6–9 months) during treatment. In addition, the current conventional TB drug therapies have shown poor ability to target and penetrate monocytes and macrophages [16,17]. Since Mtb resides in host macrophages, TB drug delivery systems that are capable of targeting the dwelling Mtb in the macrophages are highly desirable. The selectivity and targeted delivery of drugs to the monocytes/macrophages will not only reduce dosage but can also reduce toxic side effects and shorten treatment duration.

The recruitment and movement of the monocytes across blood vessel to the site of injury, infections and then to draining lymph nodes [15,18] makes them a very useful therapeutic target which can be exploited in the treatment of infections such as tuberculosis [19,20], cancer [21,22] as well as HIV [23]. Isoniazid (INH) is a first-line drug used worldwide for treating TB and possesses powerful bacteriostatic properties [24]. It acts by blocking the synthesis of mycolic acid [25] in Mtb cells. However, one of the major drawbacks of the conventional INH dosage forms is their poor cell targeting ability [16,19].

Several approaches using different drug delivery systems such as liposomes [26,27], micelles [28,29], mesoporous silica nanoparticles [30,31], solid lipid nanoparticles [32,33] and poly(lactic-*co*-glycolic acid) (PLGA) microparticles [34] have been reported for the efficient delivery of isoniazid. These nanosystems demonstrated better efficacy and other advantages over the administration of pure INH [35]. However, the use of the liposome delivery system was associated with some drawbacks including stability problems, especially when stored for a long time at room temperature due to aggregation or agglomeration as well as low encapsulation efficiency [26,27]. Also, Hwang et al. reported the design of mesoporous silica nanoparticles coated with poly(ethylene imine)-poly(ethylene glycol) (PEI-PEG) for the delivery of INH to infected TB macrophages [30]. However, the method of synthesis for this prodrug and subsequent PEI-PEG coating in this work involves the use of multiple components, lengthy reaction time and four separate chemical reactions [30]. Additionally, the use of PEI can be highly toxic [36,37]. Furthermore, the safety data and long term toxicity studies for some of the nanoparticles in use is still in doubt [38]. For example, the popular ‘stealth’ coatings of PEG may result in slow excretion from the body due to accumulation of high molecular weight PEG polymer in the liver, which can lead to macromolecular syndrome [39]. In contrast, inulin is already approved as a pharmaceutical excipient with Food and Drug Administration (FDA) Generally Recognized As Safe (GRAS) status. Moreover, this insoluble semi-crystalline form of inulin also possesses potent adjuvant activity [40,41,42] and displays no evidence of toxicity [43].

Therefore, the design of a drug-delivery system that can target intracellular pathogen in their subcellular compartment and release TB drug cargo.This ultimately can help overcome the problem associated with the pathogen using monocytes/macrophages as a safe heaven. Delta inulin or Advax™ has unique immunomodulatory properties that make it very useful as a vaccine adjuvant for viral, bacterial, protozoan antigens, as well as in anticancer treatments [42,44,45,46,47,48]. To expand the potential use of delta inulin as a drug delivery platform, we hypothesized that inulin microparticles may promote passive homing of drugs to the monocytes for targeted drug delivery in TB. This is based on well-documented use of microparticle conjugates for monocyte-mediated delivery [49,50]. Furthermore, previous work that shows delta inulin is efficiently internalized by monocytes [51] and suggests it could work as a conjugate to deliver isoniazid to Mtb infected cells. To achieve delta inulin-based drug delivery of INH, we have constructed a pH-sensitive conjugate (Inulin-INH) system by covalently modifying the inulin microparticles with biolabile hydrazone linker to INH. Upon internalization into monocytes, the linkages between inulin microparticles and the drugs are degraded by the acid conditions and enzymes contained in the lysosomes. This results in a targeted and controlled release of the drug inside the infected monocytes. The drug-modified inulin particles were characterized using Fourier transform infrared spectroscopy (FTIR) and proton nuclear magnetic resonance (^1^HNMR) drug release and then testing for cellular uptake and cytotoxicity. This inulin drug delivery system presents a unique opportunity for targeted delivery of drugs to monocytes combining positive attributes including simple chemical modification, cost-effective processing, scalable production, and low toxicity.

## 2. Materials and Methods

### 2.1. Materials

The inulin particles were supplied by Vaxine Pty Ltd. (Adelaide, Australia) and prepared using a previously described method [52]. The inulin particles are made exclusively from very short polymer chains typically isolated at ~40 fructose units and up to 100 units by the annealing conditions [53]. The microparticles are normally between 1 and 2 μm diameter [49,51,54].

Analytical grade of sodium metaperiodate, isoniazid, sodium acetate, glycerol, sodium cyanoborohydride solution (5 M in 1 M NaOH), phosphate-buffered saline tablets (PBS, pH 7.4, 0.01 M), fluorescein-5-thiosemicabizide, 10% albumin-dextrose-catalase and 0.1% Triton X-100 were all purchased from Sigma Aldrich (Castle Hill, New South Wales, Australia). Solvents for NMR experiments deuterated dimethyl sulfoxide (DMSO-*d*_6_) and deuterated water (D_2_O) was purchased from Cambridge Isotope Laboratories (Tewksbury, MA, USA). CelluSep T4 25-mm flat width 12,000 MWCO Dialysis membranes were purchased from Fisher Biotec Australia. RAW Cell 264.7 macrophage cells were provided by Vaxine Pty Ltd. (Adelaide, Australia). Cell culture reagents including Dulbecco’s Modified Eagle Medium (DMEM), l-Glutamine, trypsin and McCoy’s 5A (Modified) Medium were all purchased from Thermo Fisher Scientific (Thebarton, Adelaide, Australia), Mtb strain H37Rv from BEI Resources (Manassas, VA, USA) and Middlebrook 7H11 agar (Sparks, MD, USA). High purity water obtained from Sartorius™ Arium pro Ultrapure Water System (Göttingen, Germany) operating at 18.2 MΩ was used throughout the experiment.

### 2.2. Oxidation of Inulin Particles

The oxidizing agent sodium periodate (40 mg) was dissolved in sodium acetate buffer (0.1 M, pH 5.5, 4 mL) in a falcon tube which was protected from light. In a separate light protected falcon tube, inulin particles (3.4 mL, 53 mg/mL) was added to acetate buffer (10.7 mL). Then, the dissolved sodium periodate solution (3.92 mL) was added to the inulin dispersion and both were reacted at room temperature in the dark for 45, 60 and 90 min to allow oxidation of the inulin particles. The reaction was stopped and then quenched with glycerol (1.8 mL) before centrifuging (rcf = 3270 for 7 min). The oxidized particles were washed three times with PBS (rcf = 3270 for 7 min) and the final oxidized particles were redispersed in PBS.

### 2.3. Isoniazid (INH) Coupling to the Oxidized Inulin Particles

INH (9.6 mg) was added to oxidized inulin particles (45 mg) dispersed in PBS buffer (4.5 mL). The mixture was stirred at room temperature with varying reaction times (3 h, 6 h, and 24 h). The inulin particles were then washed three times by centrifuging (rcf-4500 for 2 min) and the supernatant replaced by fresh PBS.

### 2.4. Characterization of Inulin-INH

#### 2.4.1. Nuclear Magnetic Resonance (NMR)

The modification and oxidation of vicinal hydroxyl groups in inulin into aldehyde groups and the successful covalent attachment and loading of INH to the oxidized inulin was confirmed with proton nuclear magnetic resonance (^1^H NMR) spectroscopy (Bruker Avance III 500 NMR Bruker, Wissembourg, France) operating at 300 MHz. Briefly, 20 mg of the inulin particles and conjugate was washed twice in deuterated water then the resulting pellet was dissolved in 0.5 mL D_2_O by heating at 70 °C for 2 min and then a total of 64 scans was obtained for each sample.

#### 2.4.2. Fourier Transform Infrared Spectroscopy (FTIR)

FTIR spectra of the inulin conjugate and INH were recorded with a Shimadzu FTIR-8400S Spectroscope (Kyoto, Japan). Samples were prepared by adding 2 mg of the freeze-dried particles to 100 mg of pure FTIR-grade potassium bromide and the mixture was compressed into a disc with the help of hydraulic press using 8 t pressure. Spectra were recorded over a range of 4000 to 400 cm^−1^ using 32 scans with a resolution of 4 cm^−1^.

#### 2.4.3. Thermogravimetric Analysis (TGA)

Thermogravimetric analysis (TGA) data for samples were obtained using a Thermogravimetric Analyzer Discovery TGA 550 (New Castle, DE, USA). For the TGA experiment, approximately 6.0 mg of the sample (inulin-INH, inulin, and INH) was heated under a flow of nitrogen gas at a heating rate of 10 °C/min from room temperature to 600 °C

#### 2.4.4. Differential Scanning Calorimetry (DSC)

Differential scanning calorimetry (DSC) was performed using Discovery DSC 2920 from TA Instruments (New Castle, DE, USA) calibrated with an indium standard. The synthesized conjugate, INH and inulin were weighed (2.0 ± 0.5 mg) accurately and placed in the aluminum pans and heated from room temperature to 250 °C under a nitrogen atmosphere with a heating rate of 10 °C/min.

#### 2.4.5. X-Ray Powder Diffraction (XRD)

The X-ray powder diffraction (XRD) pattern of the samples (synthesized conjugate, inulin microparticle, and pure INH) was measured using a Empyrean, Malvern Panalytical XRD equipment (Empyrean XRD Worcestershire, UK) coupled with a graphite crystal monochromator with filter radiation of Cu-Kα1 (λ = 1.5406 Å) at 30 kV and 30 mA. The diffraction angle for the analysis (2θ) was from 5° to 50° with a scanning speed set at 1.2°/min in order to accurately measure the crystallinity of the sample.

#### 2.4.6. Scanning Electron Microscopy (SEM)

Scanning electron microscopy (SEM) imaging of the unmodified inulin microparticles and the conjugate was investigated using scanning electron microscopy Zeiss Merlin Field-Emission Gun with silicon drift detector Energy-Dispersive X-Ray Spectroscopy (SDD EDS) (Jena, Germany). Prior to the analysis, the samples were freeze-dried and subsequently placed on a double-sided tape. Finally, this was sputter-coated with platinum and then examined using accelerating voltage in the range of 2–5 KV.

#### 2.4.7. Zeta Potential

The zeta potential of inulin and the conjugate was carried out at room temperature using a Malvern Zetasizer Nano ZS instrument (Malvern, Worcestershire, UK) equipped with a 532 nm laser and at a fixed scattering angle of 173°. In brief inulin and the conjugate were washed in water three times then it was redispersed in pure water to get a concentration of 0.1 mg/mL before measuring the zeta potential. The zeta potential (mV) was calculated from the electrophoretic mobility using the Smoluchowski relationship and assuming that K · a ≫ 1 (where K and a are the Debyes–Hückel parameter and particle radius, respectively).

### 2.5. INH Loading Content Determination

For the quantification of INH content in the delivery system, a previously reported literature method with slight modification was used [55]. Inulin-INH (20 mg) was added to sodium acetate buffer (pH 3.6) and then stirred at 50 RPM for 24 h. The suspension was then centrifuged for 5 min (4500 rcf) and the supernatant was filtered before determination of the INH content using high-performance liquid chromatography (HPLC). The HPLC method was a reversed phase method using ZORBAX Eclipse Plus analytical column (RP-C18, 5 μm, 4.6 mm ID × 150 mm) connected to an HPLC system from Shimadzu Corporation (Shimadzu Corporation, Kyoto, Japan) consisting of a degas system, of a series of LC-20ADXR pumps, SIL-20ACXR autosampler, CTO-20AC column oven and SPD20A ultraviolet (UV) detector as well as CBM-20AC communication module. The mobile phase was a mixture of monobasic sodium phosphate (0.01 M) and acetonitrile (95:5 *v*/*v*) eluted at a flow rate of 1.0 mL/min and wavelength of 254 nm was then selected for UV detection of the INH peak. The run time for each sample was set at 6 min. Pure INH standard was used to plot a standard linear calibration curves (R2 ≥ 0.9989) over the range of 0.05–10 μg/mL. For all assay suitable dilution was done to ensure it fall within the calibration curve.

### 2.6. Release of INH from the Synthesized Conjugate

The in-vitro studies were carried out using media that mimic both the lysosomal environment artificial lysosomal fluid (ALF) and simulated body fluid (SBF) [56]. Also, both media were prepared using a method reported in the literature [56]. About 20 mg of the freeze-dried inulin conjugate was reconstituted with 2 mL of release media and this was transferred directly into a dialysis bag with 12,000 Da molecular weight cut off (MWCO). Thereafter, this was immersed into 10 mL of the release media; ALF at pHs 4.5, 5.2 and 6.0 and SBF at pH 7.4. The composition and preparation of ALF and SBF supplied in the supplementary section (Tables S2 and S3).

The temperature was maintained at 37 °C and stirred continuously at 50 revolutions per minute (RPM). At specific time intervals, 1 mL was collected from the outside of the dialysis bag and replace with an equal volume of fresh media. Finally, the amount of INH cleavage from this conjugate delivery system was assayed with HPLC using the same method as described above.

### 2.7. Efficient Uptake of Fluorescein-5-Thiosemicarbizide (F5TSC) Labelled Inulin Particles by RAW 264.7 Cells

Aldehyde functionalized inulin microparticles were labeled with fluorescein-5-thiosemicarbizide (F5TSC) using similar conjugation methods as INH protocol. This coupling reaction results in the formation of the inulin-F5TSC conjugate (INUF5TSC). For internalization of the INUF5TSC, RAW 264.7 cells of approximately 1 × 10^5^ cells/well were plated into each 12-well plate. To promote proper attachment to the plates, the cells were left overnight at 37 °C, 5% CO_2_, in high glucose media. Subsequently, the cells were exposed to the labeled inulin conjugate (INUF5TSC) as well as free pure F5TSC at a concentration of 20 µg/mL for 0.5, 2 and 4 h. The cells were then thoroughly washed three times using sterile PBS, then scrapped off followed by centrifugation for 5 min at 1000 rpm. The pellets were then suspended in about 100 µL of PBS. Cytospin4 cytocentrifuge (Thermoshadon, Cheshire, UK) was used to ensure uniform and even cell distribution prior to staining by spinning down at 1000 rpm for 5 min. The slides were then allowed to dry for about 24 h before fixation for another 10 min in neutral buffered formalin. Counterstaining and coverslipping were then performed using 4,6-diamidino-2-phenylindole (DAPI) containing fluorescent mounting media. Lastly, all the images were taken using the Zeiss Elyra PS.1 A laser-scanning confocal microscope (CLSM) with Zeiss ZEN lite software (Carl Zeiss, Jena, Germany).

### 2.8. Flow Cytometry Measurements (FACS)

RAW 264.7 cells of ~1 × 10^6^/mL were used to seed 12-well plates and proper attachment promoted by incubating for 24 h at 37 °C, 5% CO_2._ Then the plating media was replaced with a media containing either F5TSC (20 µg/mL) or the INU F5TSC conjugate at a final concentration of 20 µg/mL equivalent of F5TSC and these were incubated at 37 °C for 30 min, 2 h, and 4 h. At the end of each time point, the cells were rinsed and washed with PBS three times, harvested by scraping and centrifuging at 1000 rpm for 5 min. The supernatant was discarded while the pellets were resuspended with 200 µL of PBS. The percentage of the RAW 264.7 cells containing F5TSC was analyzed using CytoFLEX^TM^ flow cytometry (Beckman Coulter, CA, USA) and the data were analyzed with FlowJo^TM^ software (Becton, Dickinson and Company (BD), Warwick, RI, USA).

### 2.9. Intracellular Antibacterial Assay

The antimycobacterial effect of the inulin INH conjugates and pure INH sample against intracellular Mtb residing in macrophages was evaluated. Mtb strain (H37Rv) were cultured in Middlebrook 7H9 broth supplemented with 10% albumin-dextrose-catalase. We seeded 1 × 10^5^ RAW 264.7 cells/well in a 96 well plate with RPMI 1640 containing 10% FBS (at 37 °C containing 5% CO_2_) and infected with Mtb at an MOI of 1 for 4 h. Then, the extracellular bacteria were removed by washing with fresh media. Different concentrations (1, 10, 77 and 100 µg/mL) of the conjugates and free INH controls were subsequently added to the infected cells and the plate was incubated for 5 days. At 5 days post-infection the cell supernatants were discarded and the treated cells were lysed with 0.1% Triton X100, plated on Middlebrook 7H11 agar and cultured at 37 °C for 2–3 weeks. At the end of three weeks, colony-forming units (CFU) were counted and the results were expressed graphically using origin software.

## 3. Results and Discussion

### 3.1. Isoniazid Coupling to Delta Inulin Particles

**Figure 1:**
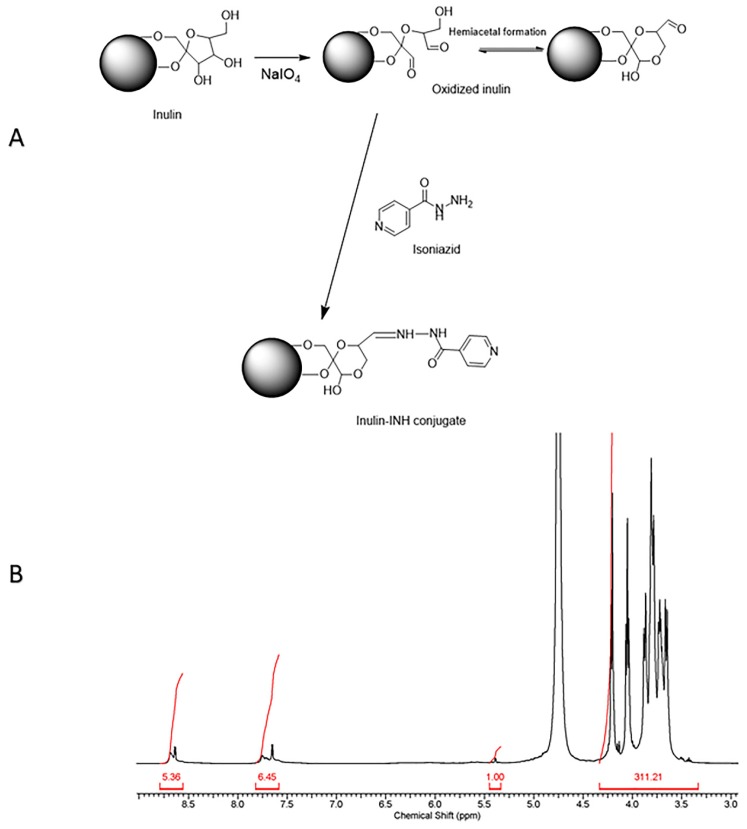
(**A**) showing the oxidation of inulin and coupling of isoniazid to inulin particles (**B**) ^1^H nuclear magnetic resonance (NMR) spectrum of inulin conjugate confirming isoniazid (INH) attachment.

Periodate oxidation of polysaccharides such as inulin [57], dextran [58], starch [59], and cellulose [60] allow the covalent attachment of amine to form acid-labile Schiff-base linkages. The conjugation of INH to inulin was a two-step reaction process. First of all, the vicinal hydroxyl groups on inulin were oxidized using sodium periodate resulting in the formation of a very reactive hemiacetal aldehyde derivative [61]. The second step was the conjugation of the INH hydrazide group to the oxidized inulin aldehyde group via hydrazone linkage [62]. (Figure 1A) Figure S1 shows the ^1^HNMR spectrum of raw inulin particles and is characterized by peaks corresponding mostly to fructose groups from 3.60 to 4.25 ppm and glucose H-2 and H-4 protons between 3.4 and 3.6 ppm as well as the anomeric proton peak at 5.4 ppm. In contrast, after periodate oxidation (Figure S2), there was evidence of new peak between 4.8 and 5.00 ppm and at 5.63 ppm which presumably can be attributed to the introduction of hemiacetal formation after oxidation of the inulin particles [63,64].

### 3.2. Characterization of Inulin Isoniazid Conjugate Using Proton Nuclear Magnetic Resonance (^1^H NMR)

Proton nuclear magnetic resonance (^1^H NMR) spectroscopy was used to validate the successful attachment of INH to the oxidized inulin. The spectrum of the inulin-INH derivative displayed the presence of isoniazid aromatic rings in the range 7.8–9 ppm (Figure 1B). In addition, there was evidence of a decrease in the peak intensity for the proton corresponding to the hemiacetal group the final inulin-INH conjugate. This suggests that the reaction of the aldehyde functional group with hydrazide portion of the isoniazid resulting in the formation of hydrazone bond.

### 3.3. Effect of Oxidation Time and Reaction Parameter on Loading

The influences of the different variables on the INH loading, such as oxidation time and INH coupling time to the oxidized inulin, was assessed. From Table S1, INH loading determined using the HPLC method increased both with an increase in oxidation time and time of INH coupling. This resulted in a maximum loading of 12.87% (*m*/*m*) with oxidation for 90 min and 24 h of INH coupling. Clearly increasing the oxidation time results in more aldehyde functional group which translates to higher INH attachment. However, in order to minimize the damage to the lamella nanostructure of inulin particles [63], the oxidation reaction was pegged at 90 min. This is because the nanostructured surface of delta inulin particles is fundamental for interaction with the immune system, binding, and uptake by monocytes [65] since dissolved inulin has no adjuvant properties [66]. The covalent attachment of INH to the nanostructure inulin microparticles provides stability and also prevents leaching. The loading result from this work 12.8% was slightly better or comparable with previous works from Hwang et al. with 11.3% and INH loaded poly (dl-lactide-*co*-glycolide) with 10%–11% [34].

### 3.4. FTIR

**Figure 2:**
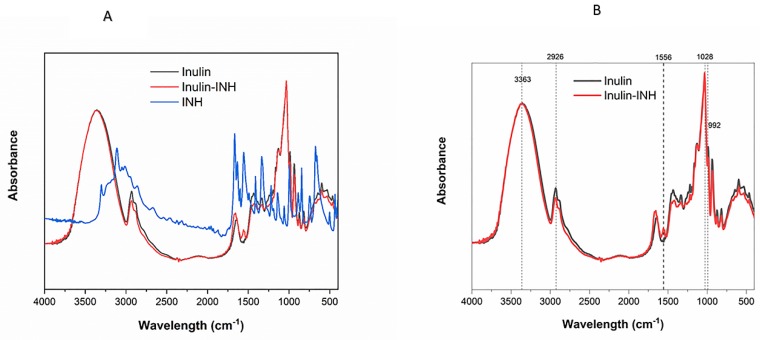
Fourier transform infrared spectroscopy (FTIR) spectra of inulin, conjugate, and INH (**A**) and inulin and conjugate (**B**).

FTIR spectroscopy further confirms the successful attachment of INH to the oxidized inulin particles. The FTIR spectra of oxidized inulin, INH and the conjugate are shown in Figure 2A,B. As reported previously, inulin particles show strong characteristic band at 3363, 2926, 1028 cm^−1^ that can be attributed to the OH stretching, aliphatic CH_2_ stretching and COC bending as well as sharper bands at 880 cm^−1^, 935 cm^−1^, 992 cm^−1^, 1037 cm^−1^, 1120 cm^−1^ and 1143 cm^−1^ that differentiate this semicrystalline inulin from raw amorphous inulin [54]. Also, the spectra of INH contains various characteristic bands of its functional groups at 3112, 1668, 1556, 1334 and 1220 cm^−1^ [67,68] (Figure S5). The appearance of clear new absorption bands in the spectrum for Inulin-INH at 1556cm^−1^ is attributable to the isoniazid pyridyl ring C=N ring symmetrical stretching [68,69] and so demonstrates the presence of INH in the final conjugate. The FTIR result of the conjugate further supports the ^1^HNMR evidence of INH conjugation to the oxidized inulin particles.

### 3.5. XRD of Inulin-INH Conjugate and Inulin Microparticles

**Figure 3:**
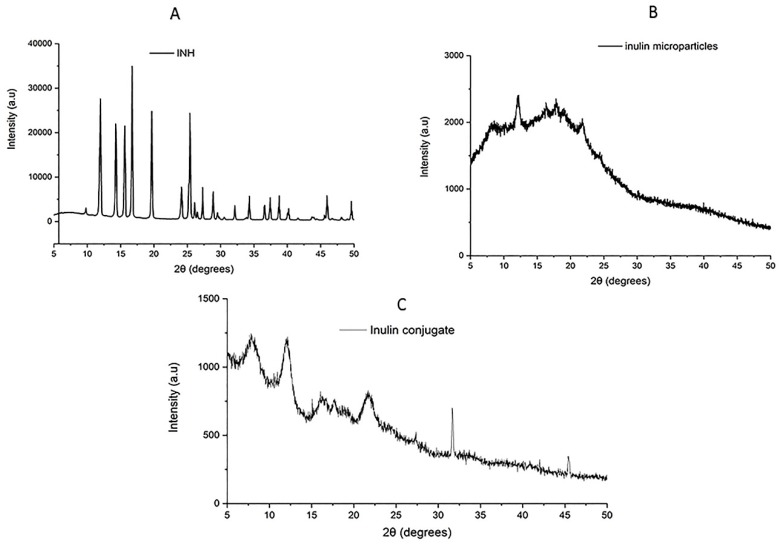
X-ray powder diffraction (XRD) diffraction graph of INH (**A**), inulin (**B**), and conjugate (**C**).

The crystallinity of the inulin conjugate and attachment of INH to the microparticles was investigated using XRD analysis. Figure 3A–C show the X-ray diffraction for both the conjugate, INH and the inulin microparticles. INH display several characteristic peaks at 2*θ* = 12 to 50° similar to previously reported diffraction result [70,71]. Inulin microparticles owning to its semi-crystalline nature display peaks at 2*θ* = 12,16.4, 17.7, 21.4, 23.8 as expected [72]. Interestingly, after the modification and conjugation of the microparticles, there was visible change to the semi-crystalline nature of the inulin. The conjugates show presence of few new peaks and broadening of existing inulin peaks. There are few peaks of the solid INH in the conjugate further confirming results by ^1^HNMR and FTIR spectroscopies. This possibly suggests that INH has been attached to the oxidized inulin producing a new conjugate [73,74].

### 3.6. Scanning Electron Microscopy (SEM)

**Figure 4:**
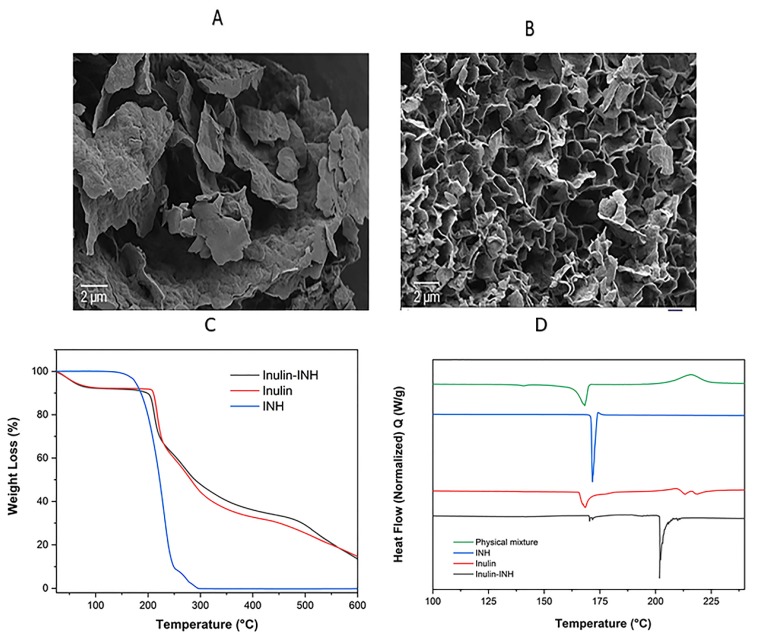
Scannng electron microscopy (SEM) micrograph of inulin microparticles (**A**) and inulin-INH (**B**). Magnification was 10,000× for both image A and B, TGA thermogram of INH, inulin microparticles and conjugate (**C**) and differential scanning calorimetry (DSC) thermograms of the physical mixture of oxidized inulin and INH, pure INH alone, inulin microparticles and conjugate (**D**).

The change in morphology after the conjugation was examined using SEM (Figure 4A,B). The micrograph images from Figure 4A,B reveals that freeze-dried inulin particles comprised of flake-like lamella sheet and spreading out at the edge as previously reported [52,54]. However, in contrast to the inulin particles the micrograph of the conjugates after modification shows a typical porous structure connected to each together due to the conjugation reaction and hydrazone bond. As expected, the two-step reaction process (oxidation reaction followed by conjugation with INH) can possibly change the well-organized nanostructured layers and arrangement of inulin particles which is very critical for the immunomodulatory properties [65]. Despite the change in morphology, the conjugates still deliver drugs to the infected monocytes suggesting that the change in morphology didn’t affect targeting efficiency. Secondly the inulin sample and conjugate were freeze-dried which can possibly change the morphology considering that the water of crystallization is removed [54].

### 3.7. Zeta Potential

Delta inulin particles have a negative zeta potential that is an aspect that stabilizes the particles and prevents aggregation [75]. In addition to stability, the zeta potential also influences the ability of the conjugates to interact with cells and macrophages. In this work, there was no change in the zeta potential of the inulin microparticles from −28.5 ± 0.72 mV for the unmodified particles to −29.0 ± 0.96 mV for the conjugates. Inulin microparticles have negative zeta potential because of the hydrogen bonding to water and deprotonation [76]. The hydroxyl groups in inulin can produce a negative charge when it dissociates from the polymer when added to water. Consequently, the oxidation of the microparticles and conjugation of INH did not cause any change in the zeta potential. This suggests the conjugate will retain the interaction and high cellular uptake into monocytic cells.

### 3.8. TGA

The thermal stability and the decomposition temperatures of the inulin particles, INH and conjugate were investigated with TGA (Figure 4C). As expected, the INH thermogram is characterized by a single major thermal event (major decomposition) starting around 170 °C and finishing around 300 °C [77]. Inulin microparticles display typical three-step mass loss as reported in the literature [49,78]. The first stage weight loss of about 7% is attributed to the loss of water residue, followed by 51% major weight loss due to the inulin decomposition. The thermogram of the conjugate shows a similar pattern to the inulin particle except that the onset of degradation was shifted from 205 °C to 200 °C. This is likely due to the oxidation of inulin and the hydrazone bond to INH, slightly destabilizing the final product [63].

### 3.9. DSC

DSC analysis was performed to gather information on the change in thermal phase transition as well as the effect of introducing INH via hydrazone bond on the melting and glass transition temperature of inulin microparticles. From the DSC thermogram (Figure 4D), INH shows a sharp single melting point endothermic peak at 171 °C without a change in the phase transition [79]. Inulin microparticles melt with a more complex endothermic peak between 164 °C and 180 °C due to the melting of various semicrystalline isoforms coinciding with the glass transition [65]. The thermogram shows the disappearance of the INH melting point endotherm and broad exothermic peak suggesting some form of physical interaction. The DSC of the inulin-INH conjugate reveals the appearance of new peak around 202 °C which is clearly absent in the pure INH and inulin microparticles. Furthermore, the disappearance of the sharp INH endothermic peak at 171 °C in the Inulin-INH confirms the formation of chemical linked INH to the oxidized inulin and possibly a small amount of supramolecular absorbed INH on the surface of the nanostructured. This result further complements the findings from FTIR and 1HNMR analysis.

### 3.10. In Vitro pH-Controlled Drug Release

**Figure 5:**
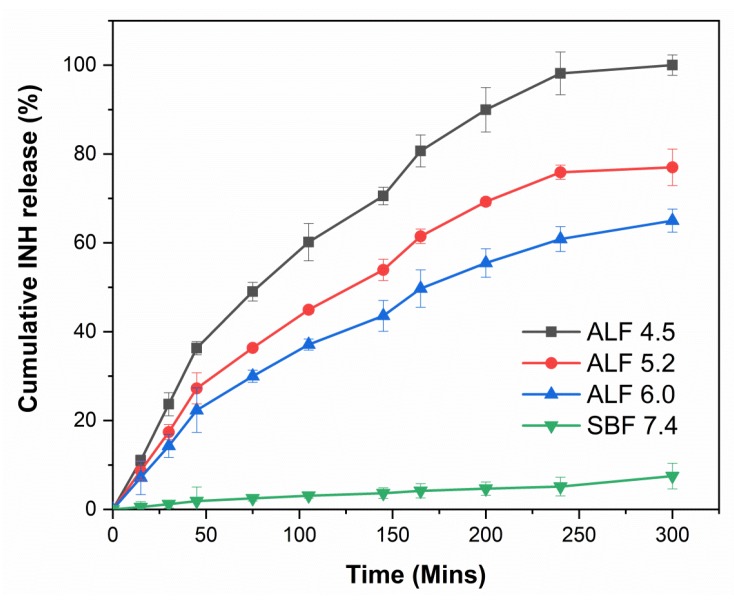
The release of INH from the synthesized conjugate in artificial lysosomal fluid (ALF) media at different pH 4.5, 5.2, 6.0 and simulated body fluid (SBF) 7.4.

The in vitro release study was carried out in order to assess and verify if the inulin conjugate was capable of allowing pH-dependent cleavage and release of INH in ALF acidic media which simulates endocytic acidic conditions and ideally less/no release in neutral physiological medium (SBF). From Figure 5, about 7.5% of INH was released after 5 h in SBF. The release of drug cargo at pH 7.4 in conjugates with a labile hydrazone bond has been reported in the past [80]. Compared with neutral/physiological pH there was a remarkable increase in INH released in ALF media. At pH 4.5, which mimics the lysosomal microenvironment, the release of INH was around 95% after 5 h. This was reduced to 77% and 65% with increasing ALF pH to 5.2 and 6, respectively. Interestingly, there was no evidence of burst release of INH, adding further evidence that the INH was covalently attached to the inulin microparticles and not part of a supramolecular mixture. The in vitro release result gives credibility to the assumption that once the conjugates are internalized into the infected monocytes, the cleavage of the biolabile linkage allows targeted and efficient release of INH into the monocytes. As expected, the pH-dependent release of INH in this work is similar to the previously reported release profile of both INH loaded liposomal and nanoparticles system [81,82]. The release profile of INH from the inulin conjugate was much better than the microparticles synthesized by other researchers which reported about 86% release in 20 min from locust bean gum particles [81]. In addition, a previous liposomal system reported drug leakage at neutral pH about 58%–64% and 22% of INH was released after 12 h [26,83] and for a polylactic acid (PLA) microparticles loaded with INH [82] burst release of about 40% was reported. In contrast, due to the covalent attachment of INH to inulin microparticles, this effect was reduced with about 7.5% of INH released after 5 h in SBF. Furthermore, the duration of release was much lower when compared to nanoparticles with PEG-PEI coating [30]. The likely reason for this can be attributed to the fact that the PEG-PEI coating resulted in better stability of the hydrazone bond.

### 3.11. Cellular Uptake of INUF5TSC by RAW 264.7 Cells

**Figure 6:**
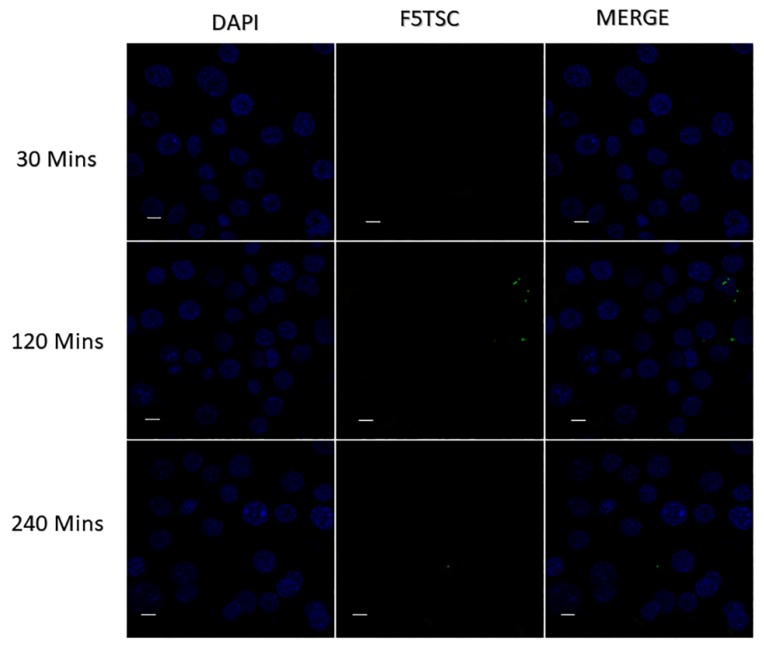
Laser-scanning confocal microscope (CLSM) image of the cellular uptake of RAW264.7 macrophage cells incubated at different time points with pure soluble F5TSC. For each panel, with the image from left to right showing cell nuclei stained by DAPI (4,6-diamidino-2-phenylindole, blue), F5TSC (green) and overlays of the two images. Scale bar = 10 µm.

**Figure 7:**
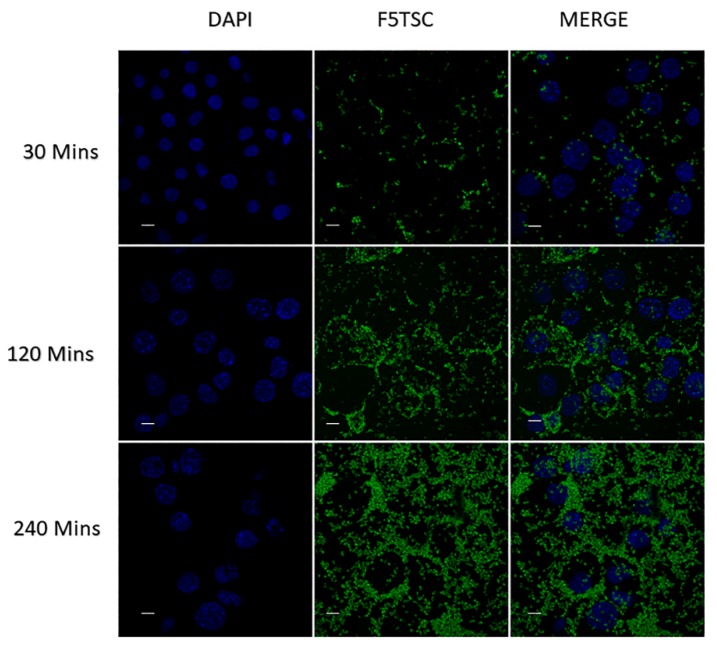
CLSM image of the cellular uptake of RAW264.7 macrophage cells incubated at different time points with F5TSC labeled inulin. For each panel, with the image from left to right showing cell nuclei stained by DAPI (blue), inulin-F5TSC (green) and overlays of the two images. Scale bar = 10 µm.

The targeting power and the excellent tropism of the conjugates towards RAW 264.7 cells were confirmed with the use of CLSM. Figure 6 and Figure 7 show CLSM images of the uptake of F5TSC and the conjugates as a function of the incubation time. It is evident that as the incubation time of the conjugated F5TSC increases from 30 min to 240 min, the fluorescence intensity also became more intense inside the RAW 264 cells. In contrast, the soluble F5TSC alone without inulin microparticles failed to show cellular uptake or accumulation in the cellular compartment. This finding thus supports earlier studies that show that monocytes exhibit a high affinity towards inulin microparticles and efficiently internalizes them [42].

### 3.12. Quantification of RAW 264.7 Cellular Uptake

**Figure 8:**
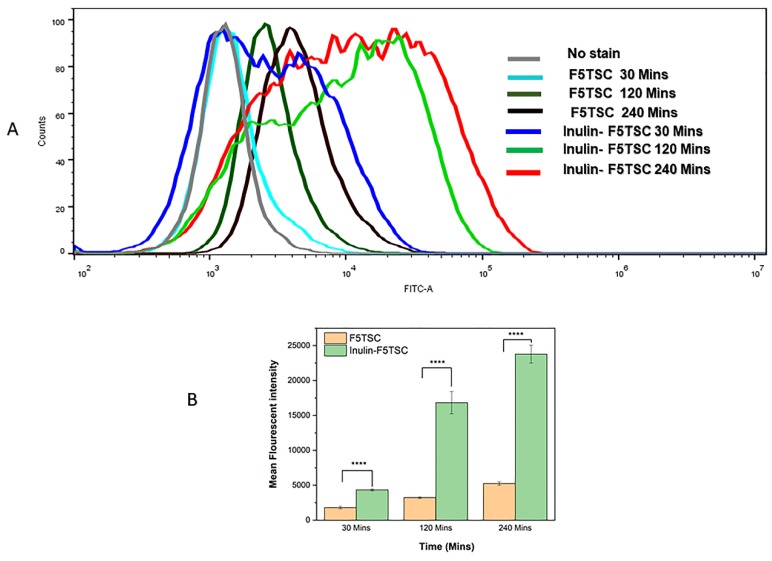
Flow cytometry analysis (histogram) of RAW 264.7 macrophage cells incubated with F5TSC-labelled inulin and F5TSC alone at different time points (30 min, 120 min, and 240 min) (**A**), the histogram of cell count versus F5TSC intensity (F5TSC -A denotes F5TSC area) showing increased uptake of Inulin-F5TSC conjugates compared with pure F5TSC by RAW 264.7 represented by increase in mean fluorescent intensity (**B**) Data is representative of three sets of experiments *n* = 3 with mean +/-standard deviation (SD) * *p* < 0.05 and **** *p* < 0.0001, ns non-significant.

Figure 8B shows the distinction in the histogram of the free F5TSC compared with conjugated F5TSC at an equivalent dose of 20 μg/mL of F5TSC in both samples. The shift in the distribution of the cell fluorescence to higher values can be ascribed to the cellular uptake of the microparticles conjugate. The fluorescent intensity of the INUF5TSC conjugate after 4 h exposure to the RAW cells 264.7 was about 5-fold greater than F5TSC alone (Figure 8B), suggesting that the conjugated F5TSC must have a different mechanism of cellular uptake. Overall, the results suggest that the inulin drug delivery system may serve as delivery vehicles for intracellular targeting of antibiotics to Mtb pathogen. A similar outcome and results were obtained in the studies conducted by other researchers [81,84,85].

### 3.13. Antimycobacterial Activity Against Infected Macrophages

**Figure 9:**
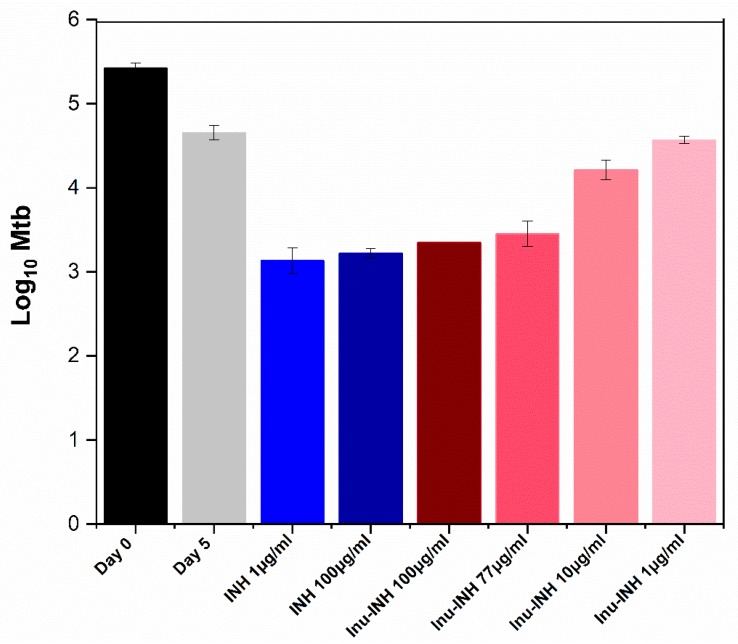
Intracellular antibacterial efficacy of pure INH and the inulin-INH conjugate suspension against *Mycobacterium tuberculosis* (Mtb) (C). Each log change represents a 10-fold difference in bacterial number. Day 0 is when the inulin-INH conjugate and soluble INH were added to the infected Mtb macrophages. Day 5 represents the cumulative drug effects after 5 days incubation.

To examine the efficacy of inulin-INH against intracellular Mtb, RAW 264.7 cells were infected with *Mtb* H37Rv and treated with different concentrations of the conjugate and free INH (Figure 9). Inulin-INH conjugate treatment displayed dose-dependent killing of *Mtb* H37Rv within RAW 264.7 cells and the conjugation of inulin to INH did not hinder intracellular efficacy. This suggests that inulin microparticles facilitate intracellular delivery of INH to infected TB pathogen as well as its strong intrinsic immunomodulatory properties against Mtb pathogen [42,52,86]. In addition, inulin-INH conjugates inhibited the growth of Mtb at a level comparable to free INH; however, inulin-INH conjugates were not as effective as free INH at lower concentrations. This may be due to different diffusion rates of INH-inulin into cells, compared to soluble INH. Thus, comparisons of the soluble drug against the inulin-INH suspension make it difficult to compare the efficacy of the targeted inulin-INH system. Both 1 and 100 µg/mL displayed similar effects (reduction of about 1.5 logs in bacterial growth when compared to untreated cells) despite an increase in the concentration of INH. A possible explanation for this could be that as INH displays efficacy against actively growing bacilli, the remaining Mtb are non-replicating bacteria population which are unaffected by the INH. A similar outcome has been reported for INH previously [87]. This outcome highlights the need for in vivo experiments to support the effectiveness of this system.

## 4. Conclusions

The potential of inulin microparticles as a drug delivery conjugate was demonstrated in this work with the conjugation of INH to the nanostructured surface of inulin microparticles via hydrazone bonds. The synthesized conjugates have pronounced pH-responsive release at acidic pH in ALF and significantly less release under physiological conditions (SBF). Moreover, the inulin-INH conjugate is internalized efficiently by macrophages and subsequently, the hydrazone biolabile linker can be cleaved in the acidic environment of the lysosomes resulting in the release of INH to the infected monocytes. This means that the delivery system provides cell-specific targeting and release of the INH into the nuclei and cytoplasm of the monocytic cells making them an effective system for targeted drug delivery in the treatment of chronic TB. Finally, the Inulin-INH conjugates exhibited intracellular *Mtb* killing effect.

## Supplementary Materials

The following are available online at https://www.mdpi.com/1999-4923/11/11/555/s1: Figure S1. ^1^H NMR spectrum of raw inulin microparticles; Figure S2. ^1^H NMR spectrum of oxidized inulin; Figure S3. ^1^H NMR spectrum of inulin conjugate; Figure S4. ^1^H NMR spectrum of pure INH; Figure S5. FTIR Spectrum of pure INH; Figure S6. FTIR Spectra of inulin and inulin-INH conjugate between 1800 and 800 cm^−1^; Table S1. shows The relationship between oxidation time and INH loading; Table S2. Preparation of SBF; Table S3. Preparation of ALF.
