# Supplementary material for: Synthesis and Characterization of pH-Sensitive Inulin Conjugate of Isoniazid for Monocyte-Targeted Delivery
Source: Pharmaceutics. 2019 Oct 28;11(11):555. doi: 10.3390/pharmaceutics11110555 (PMC6920787; doi:10.3390/pharmaceutics11110555)
Supplement: Supplementary file 1 [file pharmaceutics-11-00555-s001.pdf]

pharmaceutics

Supplementary Materials: Synthesis and Characterization of pH-Sensitive Inulin Conjugate of Isoniazid for Monocyte-Targeted Delivery

Franklin Afinjuomo, Thomas G. Barclay, Ankit Parikh, Rosa Chung, Yunmei Song, Gayathri Nagalingam, Jamie Triccas, Lixin Wang, Liang Liu, John D. Hayball, Nikolai Petrovsky and Sanjay Garg

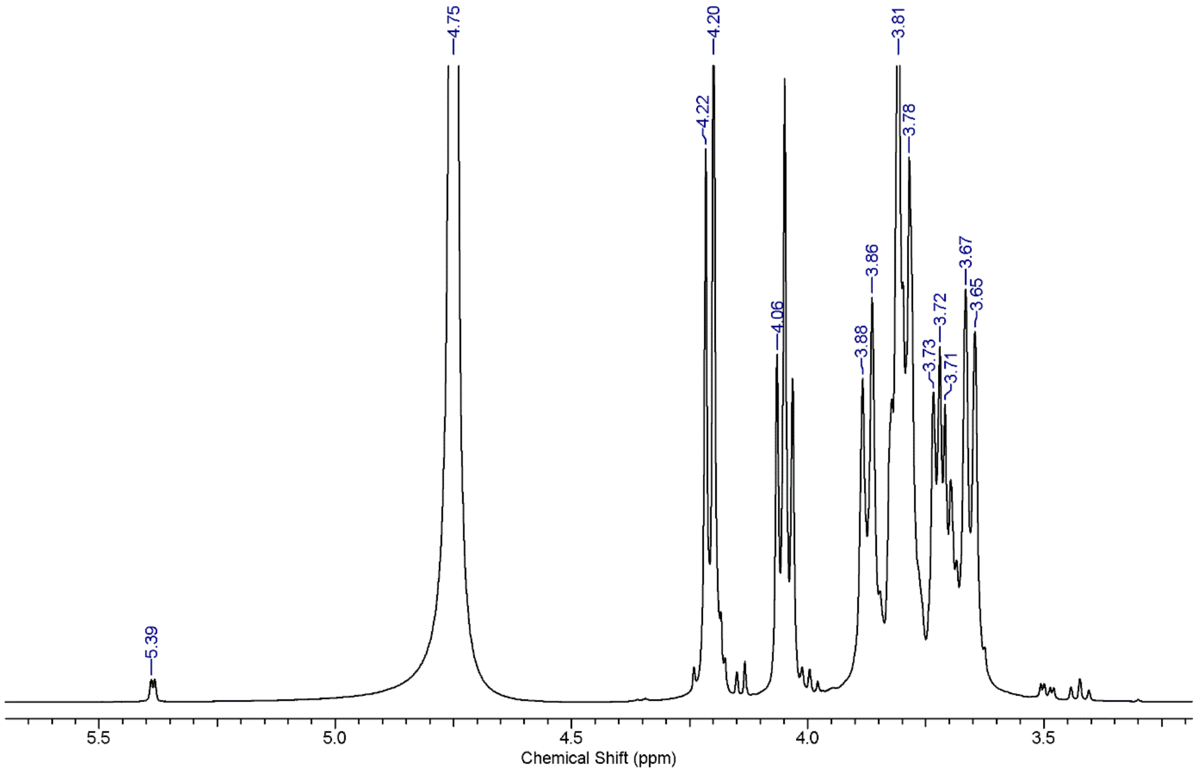

Figure S1. ^{1}H NMR of Inulin microparticle in D20 water (Peak at 4.75 belong to the solvent peak) peak at 5.39 is the glucose anomeric peak.

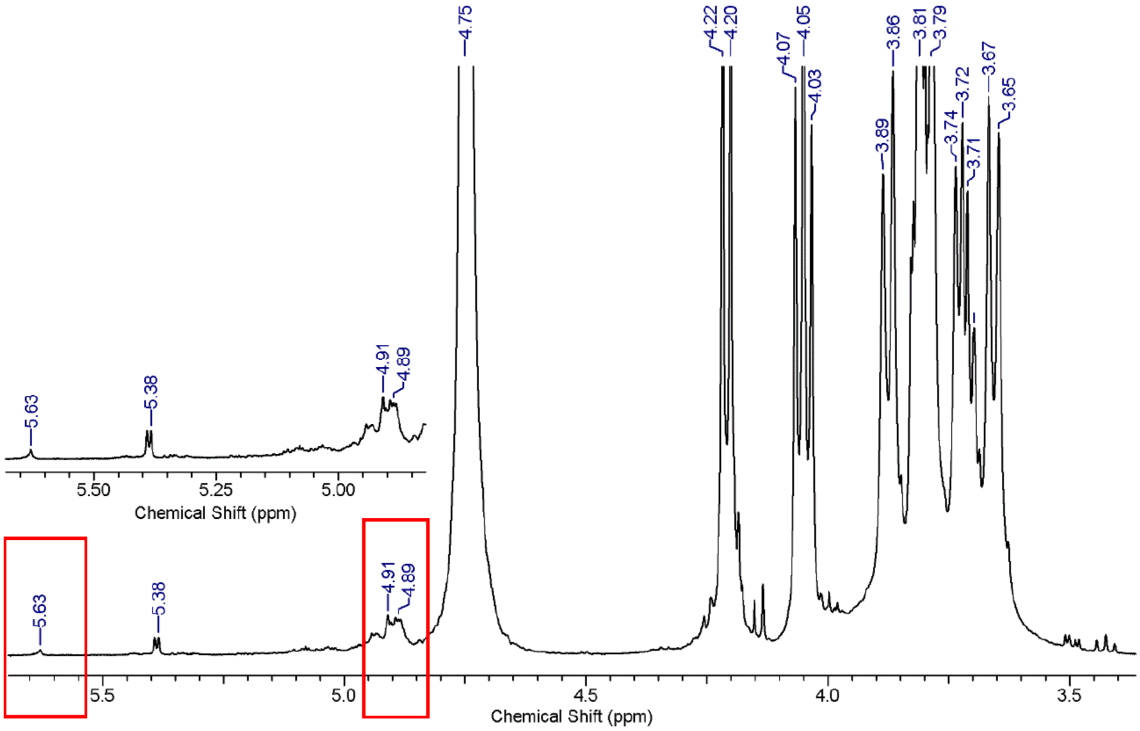

Figure S2. ^{1}H NMR of Oxidized Inulin in D20 water.

There was a slight difference between the inulin microparticles and the oxidized form from the $_{1}$HNMR result. The result shows a slight modification due to oxidation. Furthermore, after the reaction of oxidized inulin with INH there was clear evidence of reduction in the peak intensity at 5.63 and 4.89–4.91 ppm

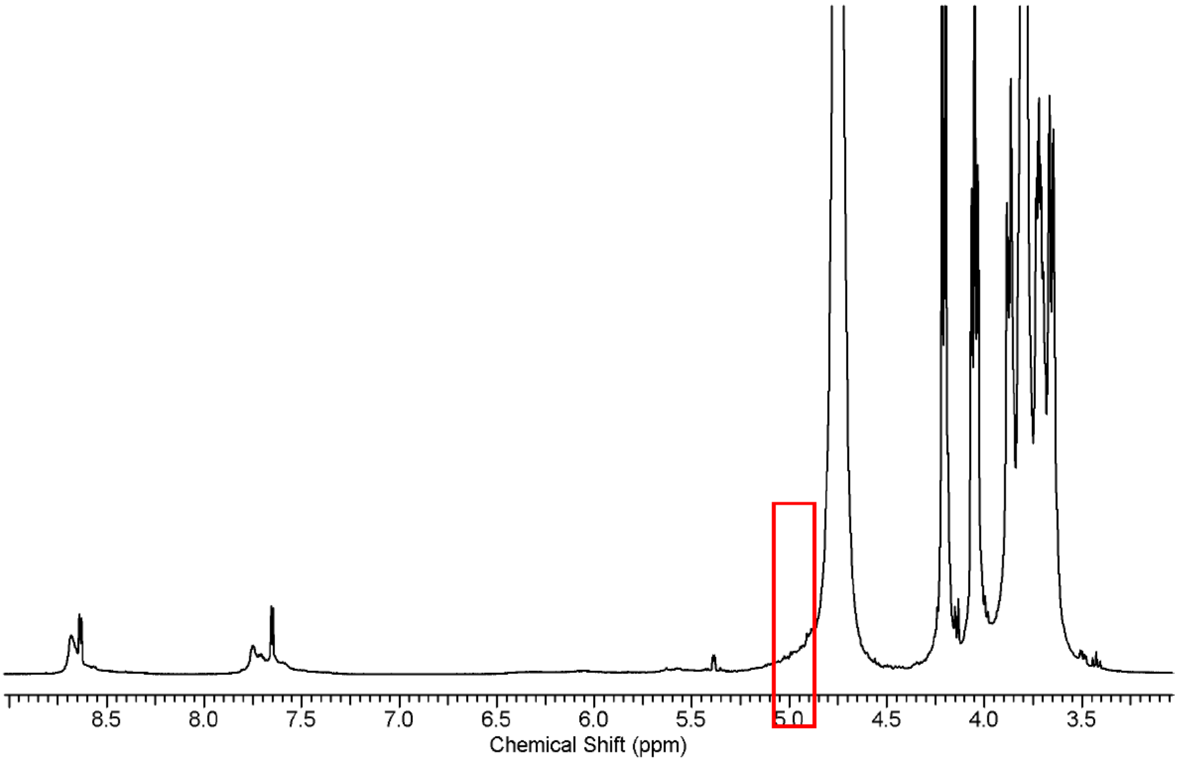

Figure S3. ^{1}H NMR spectrum of inulin conjugate.

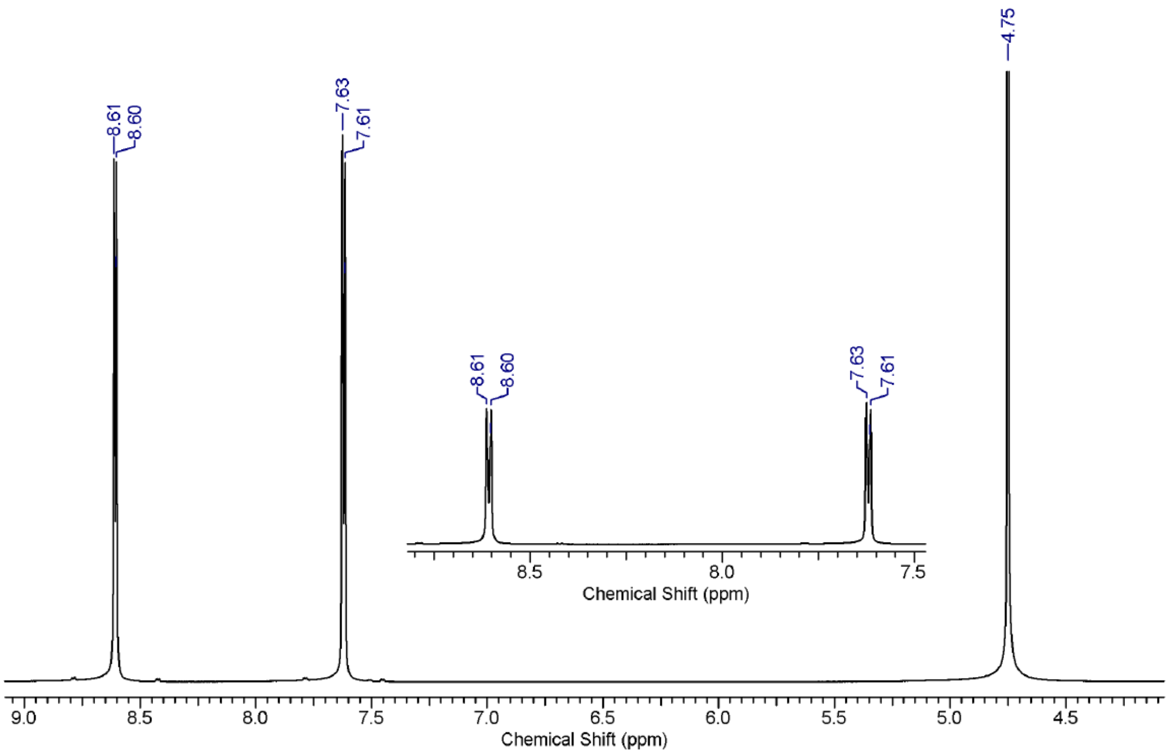

Figure S4. ^{1}H NMR spectrum of pure isoniazid.

Table S1. The relationship between oxidation time and INH loading.

Reaction Time	Oxidation 45 (% *m*/*m*)	Oxidation 60 (% *m*/*m*)	Oxidation 90 (% *m*/*m*)
INH Coupling 3 h	2.7 ± 0.3	3.94 ± 0.4	6.2 ± 0.15
INH Coupling 6 h	3.6 ± 0.5	4.83 ± 0.72	7.56 ± 0.89
INH Coupling 24 h	4.6 ± 0.25	7.32 ± 0.45	12.87 ± 0.35

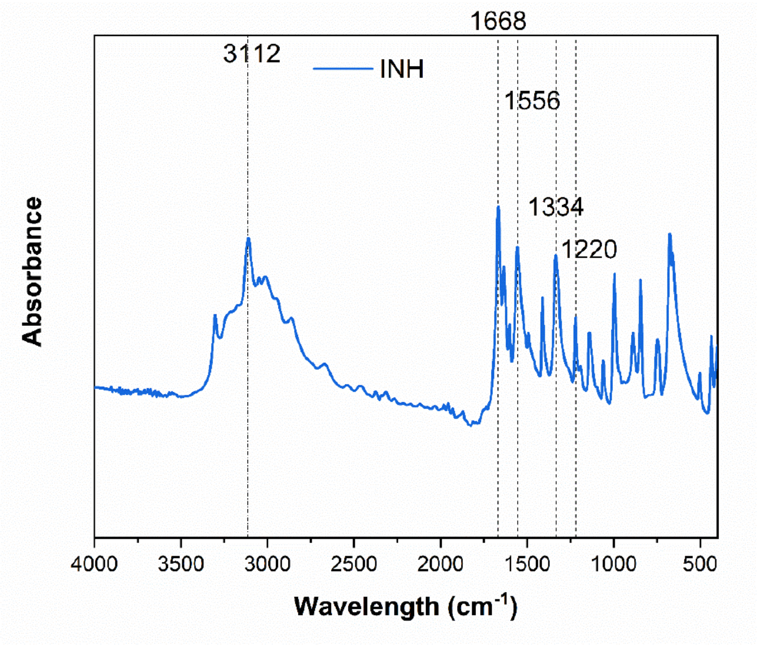

Figure S5. FTIR spectra of INH.

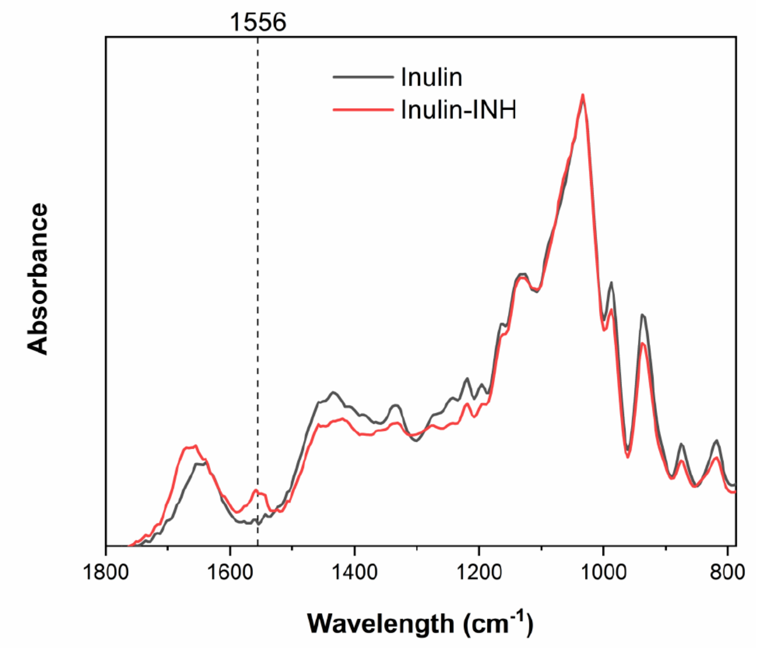

Figure S6. FTIR spectra of inulin and Inulin-INH conjugate between 1800 and 800 cm^{-1}.

The artificial lysosomal fluid was prepared from the reference [1].

Table S2. Preparation of SBF.

Material	Concentration (g/L)
Sodium chloride	8.035 g
Sodium bicarbonate	0.355 g
Potassium chloride	0.225 g
Potassium phosphate dibasic trihydrate	0.231 g
Magnesium chloride hexahydrate	0.311 g
1 M hydrochloric acid 39 mL	39 mL
Calcium chloride	0.292 g
Sodium sulfate	0.072
Tris(hydroxymethyl) aminomethane	6.118 g

Table S3. Preparation of ALF.

Material	Concentration (g/L)
Magnesium chloride	0.050
Sodium chloride	3.21
Disodium hydrogen phosphate (Na_2HPO_4)	0.071
Sodium sulfate	0.039
Calcium chloride dihydrate	0.128
Sodium citrate dihydrate	0.077
Sodium hydroxide	6.00
Citric acid	20.8
Glycine 0.059	0.059
Sodium tartrate dihydrate 0.090	0.090
Sodium lactate 0.085	0.085
sodium pyruvate 0.086	0.086

References

[1] M. Marques, R. Löbenberg, and M. Almukainzi, Simulated Biological Fluids with Possible Application in Dissolution Testing. *Dissolution Technol* **2011**, *18*, 15–28.
